# Effects of *Astragalus membranaceus* and *Panax notoginseng* Saponins Extract on the Pharmacokinetics of Whey Protein Absorption, Intestinal Permeability, and Muscle Function: A Pilot Study

**DOI:** 10.3390/nu18030504

**Published:** 2026-02-02

**Authors:** Shu Ru Zhuang, Chi-Hua Yen, Kuan-Yu Lin, You-Cheng Shen

**Affiliations:** 1 Department of Nutrition, Chung Shan Medical University, Taichung 402, Taiwan; cristine0131@gmail.com; 2 Department of Medicine, Chung Shan Medical University, Taichung 402, Taiwan; cshe190@csh.org.tw; 3 Department of Family & Community Medicine, Chung Shan Medical University Hospital, Taichung 402, Taiwan; 4 Department of Health Industry Technology Management, Chung Shan Medical University, Taichung 402, Taiwan; a0987643902@gmail.com; 5 Department of Nutrition, Chung Shan Medical University Hospital, Taichung 402, Taiwan

**Keywords:** *Astragalus membranaceus*, *Panax notoginseng*, saponins, amino acid absorption, whey protein, muscle function, intestinal permeability

## Abstract

Background/Objectives: Whether saponins aid in whey protein supplementation remains unclear. We aimed to investigate the effects of *Astragalus* and *Panax saponins* (APS) on whey protein absorption, intestinal permeability, and muscle function in healthy adults across different age groups. Methods: A randomized, double-blind, placebo-controlled crossover trial was conducted with 30 healthy participants equally stratified into three age groups (18–25, 26–59, and 60–80 years), over two phases: a single-dose trial to measure immediate amino acid absorption from whey protein and a 4-week phase combining daily supplementation with resistance training to assess long-term effects on amino acid absorption kinetics, muscle function, and gut health. Results: Immediate APS supplementation resulted in a 6.67% higher area under the curve for valine, 3.62% for leucine, and 0.15% for isoleucine, compared with the placebo. After 4 weeks, APS supplementation significantly increased the absorption of valine (14.07%) and leucine (8.34%) and improved the absorption of isoleucine (6.33%). The effects were most pronounced in older adults (60–80 years), who showed a 12.74% increase in total essential amino acid absorption. APS also caused a substantially greater increase (APS: +5.20% vs. placebo: +2.44%) in grip strength, an increase (APS: +0.85% vs. placebo: +0.68%) in muscle mass, and a reduction in blood zonulin levels (APS: −13.01% vs. placebo: −0.9%), indicating improved muscle function and intestinal barrier integrity, without adverse effects on liver or kidney function. Conclusions: APS supplementation enhances amino acid absorption from whey proteins, muscle function and gut barrier integrity, especially in older adults. These findings highlight its synergistic role in improving protein supplementation efficacy for those with age-related muscle loss.

## 1. Introduction

Nutritional interventions are increasingly being recognized as critical for optimizing metabolic health and enhancing physical performance [1,2,3]. Protein supplementation, particularly with whey protein, serves as a cornerstone strategy for supporting muscle growth, repair, and recovery [4,5]. Whey protein is highly valued for its rapid digestion and biological value, making it an excellent source of essential amino acids (EAAs) and branched-chain amino acids (BCAAs), including leucine, isoleucine, and valine [1,3]. These BCAAs play a crucial role in activating the mammalian target of the rapamycin (mTOR) pathway, a key regulator of muscle protein synthesis (MPS) [6,7,8]. Notably, leucine exerts the most potent effect on mTOR activation, promoting anabolic signaling and reducing muscle protein degradation [4,7].

Despite the well-documented benefits of whey protein, individual variability in whey protein absorption and utilization remains a challenge and is influenced by factors such as gastrointestinal health, amino acid absorption efficiency, and individual metabolic differences [9,10,11]. Addressing these variations could enhance the effectiveness of protein supplementation, particularly in individuals with higher protein demands, such as athletes and older adults [12,13,14].

To overcome these challenges, recent studies have explored strategies to enhance amino acid absorption through both nutritional and gut-targeted interventions [15,16,17]. Saponins, which are bioactive compounds derived from *Astragalus membranaceus* and *Panax notoginseng* (APS), have gained attention owing to their potential to improve nutrient absorption and overall systemic health [18,19]. These compounds have been reported to improve gut barrier function, regulate intestinal permeability by modulating tight junction proteins, such as zonulin, and upregulate nutrient transporter expression [20,21,22]. Additional studies have indicated that APS exhibit diverse pharmacological properties, including enhanced nutrient absorption, improved intestinal permeability and microbiota population, anti-inflammatory effects, and immunomodulatory activity [18,19,20,23,24]. Collectively, saponins may complement whey protein supplementation by facilitating the transport of amino acids into the systemic circulation, potentially maximizing the benefits of protein intake [22,25].

This study aims to investigate the effects of *Astragalus* and *Panax* saponin extracts on whey protein absorption, intestinal permeability, and muscle function in healthy individuals across different age groups. Using a randomized, double-blind, placebo-controlled, crossover design, this study assessed the pharmacokinetics of BCAAs, grip strength, skeletal muscle mass, and intestinal permeability marker (zonulin). To investigate the influence of age on responses, the participants were grouped into three strata according to age: 18–25, 26–59, and 60–80 years. The findings of this study contribute to a comprehensive understanding of the synergistic effects of APS and whey protein, offering insights into strategies for enhancing amino acid absorption, improving intestinal permeability, and supporting muscle function.

## 2. Materials and Methods

### 2.1. Participants

This study employed a randomized, double-blind, placebo-controlled, crossover design to evaluate the effects of APS saponin supplementation on the pharmacokinetics of whey protein absorption, intestinal permeability (zonulin), and muscle function. Randomization was computer-generated. The study protocol was approved by the Institutional Review Board of Chung Shan Medical University Hospital (Protocol No. CS1-23151) and registered at ClinicalTrials.gov (NCT06110260). Written informed consent was obtained from all participants prior to enrollment.

Healthy volunteers were recruited from the Chung Shan Medical University. Inclusion criteria included age between 18 and 80 years and a body mass index (BMI) between 18.5 and 24 kg/m^2^. Exclusion criteria included participation in another clinical trial within 30 days before the study; known allergy to dairy products; and diagnosis of diabetes, obesity, hypertension, and cardiovascular, hepatic, or renal diseases. Additional exclusion criteria included smoking and the use of medications, nutritional products, or amino acid supplements that could influence the study outcomes. Participants were also excluded if they failed to comply with the protocol, defined as missing more than one week of supplementation or resistance training.

A total of 30 participants were enrolled and stratified into three age groups: 18–25, 26–59, and 60–80 years (10 participants each). All participants underwent both APS and placebo interventions in a crossover design with a 4-week washout period between treatments.

### 2.2. Interventions

The APS and matched placebo capsules were provided by NuLiv Science, Inc. (Brea, CA, USA). Each APS capsule contained 50 mg of standardized extracts: *Astragalus membranaceus* saponins (10:1 hydroethanolic extract) and *Panax notoginseng* saponins (50:1 aqueous extract), with a total saponin content of ≥1.5%, with maltodextrin as the excipient. Placebo capsules contained only maltodextrin and were identical in appearance and weight. All participants consumed unflavored whey protein isolate powder (Vilson, QingHe Organic Co., Ltd., Taoyuan, Taiwan), providing 21.6 g of protein per 25 g serving, including 6.23 g of BCAAs, and 0.55 g of arginine.

### 2.3. Experimental Protocol

The study comprised two phases: an immediate supplementation trial and a long-term supplementation trial, both of which were conducted using a crossover design. A 4-week washout period was implemented between APS and placebo interventions to eliminate any carryover effects.

### 2.4. Immediate Supplementation Trial

The participants ingested one capsule of APS or a placebo at 9:00 PM the night before testing and fasted overnight for 12 h (water permitted). The following morning, fasting blood samples were collected (0 min). The participants then consumed a second capsule (APS or placebo) along with 25 g of whey protein and 250 mL of water. Postprandial blood samples were collected at 15, 30, 45, 60, 90, 120, 150, and 180 min to assess the amino acid pharmacokinetics. During the second round, participants crossed over to the alternate intervention and repeated the test.

### 2.5. Long-Term Supplementation Trial

In the long-term trial, participants received APS or placebo capsules in combination with whey protein supplementation (0.8 g/kg/day) and daily resistance training over 4 weeks. Supplements were taken twice daily: once in the morning before breakfast and once after exercise. Resistance training was home-based and consisted of five exercises (chair squats, wall push-ups, hip bridges, squat side lifts, and semi-squat arm raises), performed in three sets of 10 repetitions per exercise, with 30–60 s of rest between sets. Following the 4-week intervention, a washout period was implemented before switching groups. Fasting blood samples were collected from the participants before and after supplementation, along with a 3 h pharmacokinetic assessment. Whey protein was administered at doses ranging from 37 to 57 g (mean, 49.23 g). Participant compliance remained high throughout the study, with adherence rates of 99.42% and 94.40% in the placebo and APS groups, respectively.

### 2.6. Analytical Procedures

Plasma amino acid concentrations were quantified using a 96-well plate extraction and purification process (MSRLN0450; Merck KGaA, Darmstadt, Germany). Plasma samples were deproteinized with 6% sulfosalicylic acid solution, vortex-mixed for 30 s, and centrifuged at 12,500 rpm for 10 min. Then, 10 μL of supernatant was collected and mixed with 190 μL of internal standard solution, vortex-mixed for 30 s, and subjected to amino acid analysis.

Amino acid separation was performed on an ACQUITY Premier BEH C18 column (2.1 × 100 mm, 1.7 μm; Waters, Milford, MA, USA) using an Agilent 1290 Infinity UHPLC system (Agilent Technologies, Waldbronn, Germany). The chromatographic separation employed a binary gradient system consisting of acetonitrile with 0.1% formic acid (mobile phase A) and 100 mM ammonium formate in water (mobile phase B). The flow rate was maintained at 600 μL/min with an injection volume of 2 μL and column temperature of 35 °C.

Detection was performed using an API 4000 triple quadrupole mass spectrometer (Sciex Applied Biosystems, Foster City, CA, USA) coupled to the UHPLC system, with positive electrospray ionization being used for the MS/MS detection at a temperature of 550 °C and voltage of 5500 V for sensitive and specific amino acid quantification.

### 2.7. Muscle Function Assessment

Grip strength was measured using an EH101 electronic handgrip dynamometer (CAMRY, Zhongshan, China). One measurement was performed per hand, and the average of both readings was recorded. Muscle mass was assessed using dual-energy X-ray absorptiometry (DXA, Hologic, Inc., Marlborough, MA, USA), which utilizes two low-energy X-ray beams to measure tissue attenuation, thereby enabling the precise calculation of total body muscle mass [26]. Body weights were recorded using calibrated digital scales.

### 2.8. Intestinal Permeability Marker

Zonulin concentrations in the plasma samples were measured using a Human Zonulin ELISA Kit (E-EL-H5560; Elabscience, Wuhan, China) to evaluate intestinal permeability. Changes in blood zonulin levels were assessed pre- and post-intervention after 4 weeks of supplementation to determine the potential effects of APS on gut barrier integrity [27].

### 2.9. Liver and Kidney Safety Evaluation

To assess the safety of APS supplementation, liver and kidney function parameters, including blood urea nitrogen (BUN), serum creatinine, aspartate aminotransferase (AST), and alanine aminotransferase (ALT), were measured using an automated biochemistry analyzer (Beckman AU5800 Series, Beckman Coulter, Brea, CA, USA) following the manufacturer’s enzymatic and colorimetric assay protocols. Measurements were conducted at baseline and after each 4-week supplementation period.

### 2.10. Statistical Analysis

All statistical analyses were performed using the SPSS software (version 20.0; SPSS Inc., Chicago, IL, USA). Descriptive data were presented as mean ± standard error (SE). The Kolmogorov–Smirnov test was employed to assess data normality. A two-way repeated-measures ANOVA (Group: placebo vs. APS × Time: Week 0 vs. Week 4) was performed to determine whether any significant main effects or interaction effects were present. Hepatic and renal safety markers were analyzed using independent-samples Student’s *t*-tests for between-group comparisons and paired *t*-tests for within-group changes from baseline. Statistical significance was defined as two-tailed *p* < 0.05.

## 3. Results

### 3.1. Demographic Data of Participants

**Figure 1:**
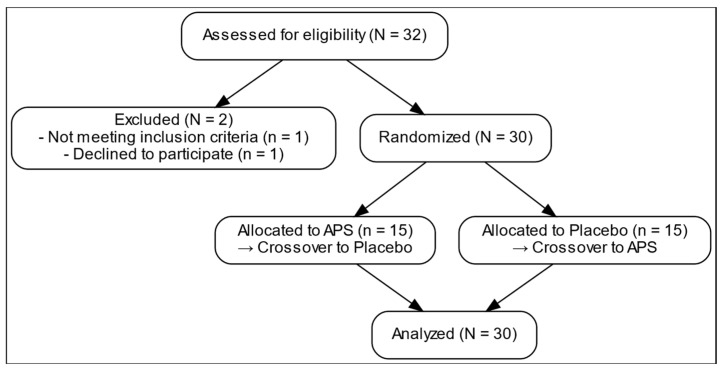
Recruitment process flow chart.

Figure 1 presents the comprehensive design of a 12-week randomized, double-blind, placebo-controlled, crossover trial examining the effects of APS extracts on amino acid absorption, intestinal permeability, and muscle function. After screening and enrollment, a placebo. 30 participants were recruited and stratified into three age groups (18–25, 26–59, and 60–80 years), with 10 participants in each group (Table 1). The overall mean age was 39.60 ± 3.27 years. The average body weight was 61.45 ± 1.80 kg, and the mean BMI was 22.14 ± 0.39 kg/m^2^. The mean height of the participants was 166.28 ± 1.79 cm. All participants underwent both APS and placebo phases in a crossover manner with a 4-week washout period. They were then randomly assigned to initial intervention sequence to receive either APS or Placebo.

### 3.2. Two-Way Repeated-Measures ANOVA Effects

A two-way repeated-measures analysis of variance (ANOVA) was conducted to evaluate the effects of APS supplementation on the pharmacokinetic parameters of branched-chain amino acids (BCAAs), including area under the curve (AUC), maximum plasma concentration (Cmax), and time to maximum concentration (Tmax). The analysis considered both the main effects of treatment (APS vs. placebo), time (immediate vs. after 4-week intervention), and their interaction (Table 2). No significant interaction effects between treatment and time were detected across any BCAA parameter (AUC, Cmax, or Tmax).

Significant main effects of treatment and time were observed for valine AUC (*p* < 0.001 for both), indicating that both APS supplementation and prolonged intake increased valine absorption. For leucine AUC, a significant treatment effect was found (*p* < 0.001), while the time effect approached significance (*p* = 0.051). No significant effects were observed for isoleucine AUC.

Regarding Cmax, valine exhibited significant treatment (*p* < 0.001) and time effects (*p* = 0.008), suggesting that APS supplementation and long-term intake contributed to increased maximum serum concentrations. However, no statistically significant differences were noted in leucine and isoleucine Cmax between treatments.

In terms of Tmax, a significant treatment effect was found for valine (*p* = 0.043) and isoleucine (*p* = 0.027), indicating faster absorption kinetics with APS. Leucine Tmax did not differ significantly between groups.

### 3.3. Effects of Immediate APS Supplementation on BCAA Absorption (Single-Dose Protein Trial)

Pharmacokinetic analysis of immediate BCAA absorption is shown in Table 3. The participants consumed a single dose of 25 g whey protein with APS or a placebo. Blood samples were collected to evaluate the BCAA absorption kinetics.

The study observed an increase in the AUC of valine by 6.67%, leucine by 3.62%, and isoleucine by 0.15% following APS supplementation compared with the placebo group. Moreover, the Cmax values of valine, leucine, and isoleucine increased by 6.27%, 8.23%, and 2.77%, respectively. Furthermore, the times to reach the maximum concentration (Tmax) of valine, leucine, and isoleucine were decreased by 9.70%, 4.44% and 6.97%, respectively. Although not statistically significant, APS supplementation led to higher AUC and Cmax values and earlier Tmax values than the placebo.

### 3.4. Effects of Long-Term APS Supplementation on BCAA Absorption (After 4-Week Supplementation)

In the long-term trial, participants received either APS or placebo capsules combined with whey protein supplementation (0.8 g/kg body weight per day) and a daily resistance training program over four weeks. Blood samples were collected post-intervention.

After 4 weeks of APS supplementation, valine AUC was significantly higher compared with the immediate value following a single dose (*p* < 0.001), suggesting enhanced absorption with long-term intake. Compared with the placebo group, the APS group showed a significantly higher AUC for valine and leucine, with significant increases of 14.07% (*p* < 0.05) and 8.34% (*p* < 0.05), respectively (Table 3). Isoleucine AUC increased by 6.33%. The Cmax value of valine increased significantly by 9.87% (*p* < 0.05) compared with the placebo, whereas that of leucine and isoleucine increased by 4.76% and 5.41%, respectively, without reaching statistical significance. In addition, compared to a single dose, 4-week APS supplementation significantly increased valine Cmax, indicating higher maximum serum concentration with long-term intake.

Compared to the placebo, the Tmax of valine and isoleucine decreased significantly by 7.14% and 12.40% (*p* < 0.05 for both), respectively. The Tmax of isoleucine occurred earlier by 2.22%.

These findings indicate that prolonged APS supplementation results in a statistically significant increase in the absorption of valine and leucine, including an enhancement in the absorption of isoleucine.

### 3.5. Effects of Long-Term APS Supplementation on Arginine Absorption (After 4-Week Supplementation)

**Figure 2:**
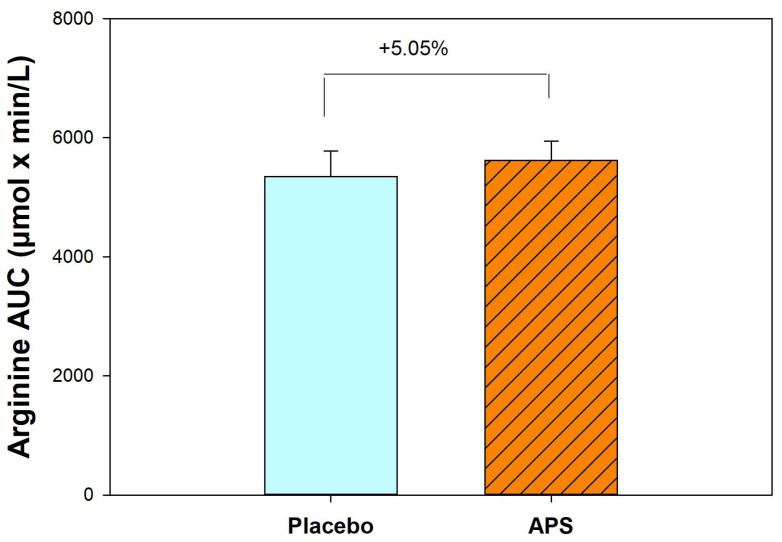
Comparison of area under the curve for arginine between APS and placebo groups (n = 30). Data are expressed as mean ± SEM.

After 4 weeks, APS supplementation resulted in a 5.05% increase in the AUC for arginine compared with the placebo group (Figure 2). These results suggest that long-term APS supplementation enhances the absorption of both BCAAs and arginine from whey protein.

### 3.6. Effects of Long-Term APS Supplementation on EAAs Absorption (After 4-Week Supplementation)

**Figure 3:**
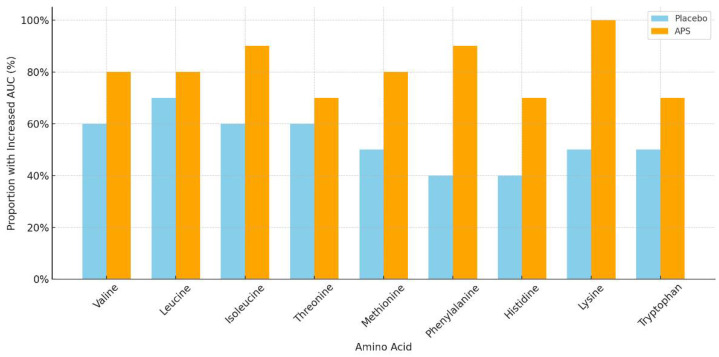
Proportion of participants aged 60–80 years with increased area under the curve for essential amino acids after long-term supplementation with APS vs. placebo.

The number of participants with increased EAA AUC levels after APS or placebo supplementation across age groups is summarized in Table 4. APS supplementation led to a higher number of individuals showing increased EAA AUC levels, particularly in the 60–80 years age group, compared with the placebo group. Specifically, the amino acids isoleucine, methionine, phenylalanine, histidine, and lysine showed markedly higher numbers of responders in the APS group (Figure 3).

**Figure 4:**
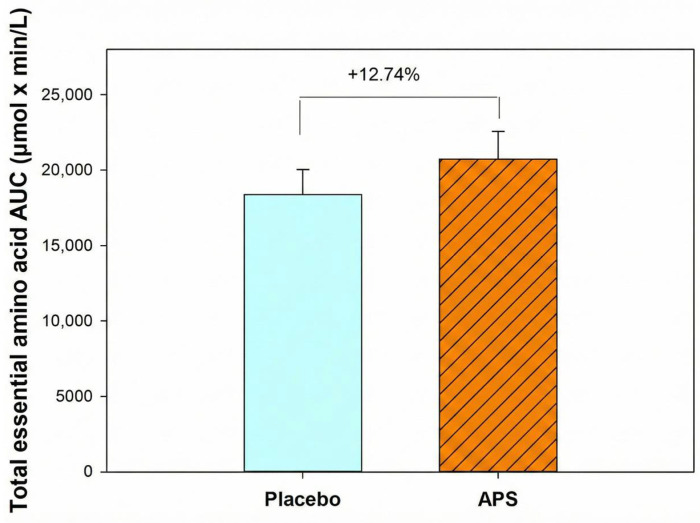
Total essential amino acid area under the curve in participants aged 60–80 years. Values are expressed as mean ± SEM.

Furthermore, participants aged 60–80 years exhibited a 12.74% higher total EAA AUC in the APS group than in the placebo group (Figure 4), indicating that APS supplementation is particularly effective in enhancing the intestinal absorption of EAAs in older adults and offers greater benefits for this age group.

Overall, these results suggest that long-term APS supplementation enhances the absorption of amino acids, not only BCAAs and arginine, but also total EAAs, from whey protein.

### 3.7. Effects of APS Supplementation on Muscle Function

The impact of APS supplementation on muscle function was assessed by measuring changes in grip strength and muscle mass over the 4-week intervention period. A two-way repeated-measures ANOVA was performed to evaluate treatment effects (APS vs. placebo), time effects (week 0 vs. week 4), and their interaction (Supplementary Table S1). Descriptive changes and between-group comparisons are presented in Table 5.

A significant main effect of time was observed for grip strength (*p* = 0.002), indicating APS improvement in handgrip strength after the 4-week intervention.

The APS group showed greater improvement in grip strength over 4 weeks (+5.20%) than the placebo group (+2.44%). Muscle mass increased by +0.85% in the APS group compared with +0.68% in the placebo group. Body weight remained stable in both groups with minimal changes (0.05% in the APS group vs. 0.37% in the placebo group). Overall, APS supplementation resulted in greater improvements in grip strength and muscle mass than placebo.

### 3.8. Changes in Intestinal Protein Blood Zonulin Levels (Intestinal Permeability)

Blood zonulin level, a marker of intestinal permeability, was evaluated at baseline and after 4 weeks. The two-way repeated-measures ANOVA results are provided in Supplementary Table S2, and the zonulin values (week 0 and week 4) are summarized in Table 6. Statistical analysis revealed no significant main effects for treatment (*p* = 0.174) or time (*p* = 0.498), and no significant interaction between treatment and time (*p* = 0.552). Although not statistically significant, APS supplementation led to a greater reduction in zonulin levels than placebo over 4 weeks.

Compared with the placebo group, the APS group showed a decrease of 13.01% over four weeks, whereas the placebo group showed only a 0.90% reduction. This represents a 13.01% reduction vs. 0.90% in placebo, indicating that APS supplementation more effectively improved intestinal barrier integrity.

Increased intestinal permeability is associated with age-related inflammation and gut dysbiosis [28]. These findings suggest that APS is a potential candidate for reducing intestinal inflammation and dysbiosis by enhancing intestinal barrier function.

### 3.9. Safety Evaluation

To assess clinical safety, clinical biomarkers of liver and kidney function were measured at baseline and after four weeks of supplementation (Supplementary Table S3). To assess clinical safety, liver and kidney function biomarkers (BUN, creatinine, AST, and ALT) were measured at baseline and after 4 weeks of supplementation (Supplementary Table S3). After 4 weeks of supplementation, BUN levels increased significantly in both the APS and placebo groups; however, all values remained within the normal reference range (7–20 mg/dL). The levels of creatinine, AST, and ALT remained stable and within the clinically accepted normal ranges in both groups.

## 4. Discussion

This study investigated the effects of APS extract supplementation on the pharmacokinetics of whey protein absorption, intestinal permeability, and muscle function in healthy adults of different age groups. The key findings indicated that immediate APS supplementation led to a 6.67% higher AUC for valine, 3.62% for leucine, and 0.15% for isoleucine, along with higher Cmax and earlier Tmax values, compared with placebo. After 4 weeks, APS supplementation significantly increased the absorption of valine by 14.07% and leucine by 8.34%, including enhancing the absorption of isoleucine by 6.33%. APS also increased arginine absorption by 5.05%. Additionally, a higher number of individuals, particularly in the 60–80 year age group, showed increased essential amino acid AUC levels, with the total EAA AUC increasing by 12.74% compared with placebo. APS supplementation also led to a substantially greater increase (APS: +5.20% vs. placebo: +2.44%) in grip strength, an increase (APS: +0.85% vs. placebo: +0.68%) in muscle mass, and 13.01% reduction in blood zonulin levels compared to a 0.90% decrease in the placebo group, indicating improved muscle function and intestinal barrier integrity.

In the immediate supplementation trial, although the AUC increased by 6.67% for valine, 3.62% for leucine, and 0.15% for isoleucine with APS supplementation, the differences were not significant. These modest short-term effects are consistent with previous reports, suggesting that single doses of herbal extracts generally produce limited immediate improvements in nutrient absorption [29,30]. This may be attributed to the time required for the mTOR pathway activation to promote intestinal epithelial cell turnover, a key mechanism underlying the regulation of nutrient transporter expression and gut permeability [18,31,32,33].

In contrast, long-term APS supplementation significantly improved the AUC for valine by 14.07% and leucine by 8.34% and enhanced the absorption of isoleucine by 6.33%, indicating enhanced intestinal absorption and systemic availability of BCAAs. This is particularly important to support the elevated protein demands of athletes and older adults [34,35,36]. These results suggest that long-term APS intake can augment and expedite amino acid uptake.

Arginine is a conditionally EAA involved in various biological processes [18,37]. Saponins from *Astragalus* have been shown to enhance arginine uptake by modulating cationic amino acid (CAT) transporters and activating the mTOR pathway [18,32,37]. Similarly, saponins improve BCAA absorption by affecting tight junction proteins and intestinal permeability [18,38]. In this study, 4-week APS supplementation resulted in a 5.05% increase in arginine AUC compared to the placebo. In contrast, our prior studies showed significant increases of 17.3% in healthy individuals and 49.7% in participants with inflammatory bowel disease, likely due to a higher arginine dose (5 g vs. 0.55 g from whey protein) and the presence of other amino acids in whey protein, such as lysine, which competes with arginine for CAT transporters and may thus result in reduced arginine absorption [32,38,39]. These factors may explain the modest arginine absorption observed in the present study.

EAAs are vital for protein synthesis and cellular repair, particularly in rapidly renewing tissues, such as the intestinal epithelium [40,41,42]. A notable finding of this study was the pronounced effect of APS in the older age group (60–80 years), in which a higher number of individuals exhibited increased absorption of EAAs. Particularly, isoleucine, methionine, phenylalanine, histidine, and lysine showed markedly higher numbers of responders in the APS group. Overall, participants aged 60–80 years exhibited a 12.74% higher total EAA AUC in the APS group than in the placebo group. The universal increase in EAAs absorption in APS-treated participants underscores its efficacy in intestinal modulation and highlights APS’s potential to counteract age-related decline in amino acid transporter function and protein metabolism [8,43,44]. These findings suggest that APS exerts broader effects on the absorption of BCAAs, arginine, and EAAs.

Efficient nutrient absorption is essential for supporting MPS and maintaining functional capacity [45,46,47,48]. Impaired absorption of amino acids may contribute to anabolic resistance, a condition in which the muscle tissue becomes less responsive to amino acid stimulation, ultimately limiting gains in muscle strength and mass [48,49,50]. Therefore, improving amino acid bioavailability using nutritional strategies may play a key role in enhancing muscle function. Regarding muscle function, the APS group suggested an improvement in grip strength after four weeks, increasing by 5.20% compared with a 2.44% increase in the placebo group. Although this difference was not statistically significant, it was likely because resistance training was performed at home by the participants without the supervision of trained professionals in a controlled environment. Furthermore, the greater increases in muscle mass (+0.85% in the APS group versus +0.68% in the placebo group) suggest that enhanced amino acid absorption may have contributed to anabolic support and improved muscle function.

Zonulin is a biomarker of intestinal permeability [27,51,52]. Beyond the effect of APS on amino acid absorption, another important finding was that APS supplementation decreased blood zonulin levels by 13.01% over 4 weeks, whereas the placebo group showed only a 0.90% reduction. This suggests improved gut barrier integrity, which could facilitate both paracellular and transcellular amino acid transport and potentially limit the translocation of pro-inflammatory macromolecules [53,54,55]. Although the between-group differences in zonulin levels did not achieve statistical significance, likely because this trial was conducted in healthy participants rather than in individuals with impaired gut function, the baseline zonulin levels were within the normal range, which may have made it more difficult to detect statistically significant changes. These results align with the previous findings that APS possesses gut dysbiosis-modulating and anti-inflammatory properties that contribute to improved barrier integrity and nutrient uptake in human participants with ulcerative colitis [19].

Collectively, these cross-population data reinforce the conclusion that APS enhances amino acid absorption in both healthy individuals and those with gut dysfunction, while improving barrier integrity and microbial composition. APS appears to exert its beneficial effects through multiple mechanisms: (1) upregulation of amino acid transporters to enhance absorption [18,19,23,31,32] and (2) improvement of gut barrier integrity via activation of the mTOR pathway to rebuild the gut epithelial cells lining. [18,19,31]. These physiological changes likely contributed to the observed improvements in muscle function, particularly grip strength, indicating that enhanced amino acid handling translates into meaningful functional outcomes. The present findings, together with the broader evidence base, suggest that APS is a safe and effective plant-based supplement that supports protein nutrition, enhances intestinal barrier integrity, and improves muscle function, particularly in older adults, individuals with age-related muscle loss such as sarcopenia, or those with compromised gut health. This study has several limitations, including a small pilot sample (n = 30), exploratory outcomes, lack of APS-only/exercise-only arms, home-based exercise without supervision, and measurement error considerations. The findings are preliminary and hypothesis-generating, and further large-scale and long-term studies are warranted to confirm these effects and elucidate the underlying molecular mechanisms.

## 5. Conclusions

The results of this clinical study suggest that supplementation with APS enhances the intestinal absorption of amino acids derived from whey protein, particularly after long-term intake. Notably, APS supplementation was particularly effective in older adults (60–80 years), indicating a 12.74% higher total EAA AUC than the placebo. Improvements were also observed in functional outcomes, with greater gains in grip strength and muscle mass and a reduction in blood zonulin levels, indicating enhanced muscle function and intestinal barrier integrity.

Altogether, APS is a safe and effective plant-based supplement for supporting protein nutrition, enhancing intestinal barrier integrity, and improving muscle function, particularly in older adults, individuals with age-related muscle loss such as sarcopenia, and those with compromised gut health.

## Figures and Tables

**Table 1:** Demographic characteristics of the study participants.

Characteristics	18–25 (Years)	26–59 (Years)	60–80 (Years)	Total
	(*n* = 10)	(*n* = 10)	(*n* = 10)	(*n* = 30)
Age (years)	22.20 ± 0.65	34.60 ± 3.12	62.00 ± 0.98	39.60 ± 3.27
Body Weight (kg)	62.21 ± 2.66	60.01 ± 3.92	62.13 ± 2.91	61.45 ± 1.80
Height (cm)	168.60 ± 2.38	165.15 ± 3.96	165.10 ± 2.96	166.28 ± 1.79
BMI (kg/m^2^)	21.85 ± 0.72	21.84 ± 0.71	22.71 ± 0.61	22.14 ± 0.39

Data are expressed as mean ± standard error (SE).

**Table 2:** Two-way repeated-measures ANOVA results for AUC, Cmax, and Tmax of BCAA [Treatment (APS vs. Placebo) × Time (Immediate (week 0) vs. Long-term (4-week) supplementation)].

	Source of Variation	F (1, 29)	*p* Value
Valine AUC	Treatment	16.042	<0.001 ***
	Time	21.654	<0.001 ***
	Treatment × Time	2.280	0.142
Leucine AUC	Treatment	21.416	<0.001 ***
	Time	4.157	0.051
	Treatment × Time	1.732	0.198
Isoleucine AUC	Treatment	1.961	0.172
	Time	2.984	0.095
	Treatment × Time	1.761	0.195
Valine Cmax	Treatment	27.906	<0.001 ***
	Time	7.977	0.008 **
	Treatment × Time	0.905	0.349
Leucine Cmax	Treatment	0.041	0.841
	Time	0.352	0.558
	Treatment × Time	1.188	0.285
Isoleucine Cmax	Treatment	2.762	0.107
	Time	0.162	0.690
	Treatment × Time	0.442	0.511
Valine Tmax	Treatment	4.474	0.043 *
	Time	0.018	0.895
	Treatment × Time	0.089	0.768
Leucine Tmax	Treatment	0.041	0.841
	Time	0.352	0.558
	Treatment × Time	1.188	0.285
Isoleucine Tmax	Treatment	5.401	0.027 *
	Time	0.986	0.329
	Treatment × Time	0.269	0.608

* *p* < 0.05; ** *p* < 0.01; *** *p* < 0.001.

**Table 3:** Pharmacokinetics of BCAA absorption (AUCx, Cmax, and Tmax) before and after 4-week APS or placebo intervention (immediate and long-term).

Variable	Treatment	Immediate (Week 0)	Long-Term (Week 4)
Valine AUC	Placebo	30,293 ± 1688	32,033 ± 1353
	APS	32,315 ± 1500	36,539 ± 1665 ***#
Leucine AUC	Placebo	34,378 ± 1672	34,625 ± 1561
	APS	35,622 ± 1580	37,512 ± 1521 #
Isoleucine AUC	Placebo	24,783 ± 1112	24,912 ± 1163
	APS	24,821 ± 1037	26,490 ± 1014
Valine Cmax	Placebo	516.97 ± 13.46	537.77 ± 13.00
	APS	549.37 ± 14.93	590.83 ± 13.45 *#
Leucine Cmax	Placebo	470.80 ± 13.20	482.07 ± 12.62
	APS	509.53 ± 15.61	505.03 ± 15.61
Isoleucine Cmax	Placebo	323.23 ± 9.78	321.50 ± 10.80
	APS	332.17 ± 10.10	338.90 ± 9.34
Valine Tmax	Placebo	67.00 ± 3.58	66.50 ± 2.76
	APS	60.50 ± 3.25	61.75 ± 2.96 #
Leucine Tmax	Placebo	67.50 ± 3.20	67.50 ± 3.28
	APS	64.50 ± 3.06	66.00 ± 3.85
Isoleucine Tmax	Placebo	61.00 ± 3.13	60.50 ± 3.17
	APS	56.75 ± 3.00	53.00 ± 2.00 #

Data are presented as means ± SE and post hoc comparisons for AUC, Cmax, and Tmax of BCAA. Asterisk indicates significant pairwise differences between immediate and long-term supplementation (* *p* < 0.05, and *** *p* < 0.001). # indicates significant pairwise differences between Placebo and APS treatment groups (*p* < 0.05).

**Table 4:** Number of participants with increased essential amino acid area under the curve levels after APS or placebo supplementation across age groups (Long-term).

	18–25		26–59		60–80	
Amino Acid	Placebo	APS	Placebo	APS	Placebo	APS
Valine	7/10	7/10	5/10	6/10	6/10	8/10
Leucine	4/10	8/10	5/10	6/10	7/10	8/10
Isoleucine	4/10	8/10	6/10	8/10	6/10	9/10
Threonine	5/10	7/10	5/10	6/10	6/10	7/10
Methionine	6/10	7/10	5/10	9/10	5/10	8/10
Phenylalanine	5/10	7/10	2/10	8/10	4/10	9/10
Histidine	4/10	4/10	5/10	7/10	4/10	7/10
Lysine	6/10	10/10	6/10	10/10	5/10	10/10
Tryptophan	3/10	6/10	7/10	7/10	5/10	7/10

Data were expressed as proportion of rise among 10 subjects.

**Table 5:** Comparative effects of APS and placebo on muscle function.

Variable	Treatment	Immediate (Week 0)	Long-Term (Week 4)
Grip Strength (kg)	Placebo	28.32 ± 1.61	29.01 ± 1.58
	APS	28.82 ± 1.42	30.32 ± 1.61 ***
Muscle Mass (kg)	Placebo	44.16 ± 1.84	44.46 ± 1.76
	APS	44.74 ± 2.03	45.12 ± 1.94
Body Weight (kg)	Placebo	61.45 ± 1.80	61.68 ± 1.81
	APS	61.57 ± 1.87	61.60 ± 1.87

Data are presented as means ± SE and comparisons for grip strength, muscle mass, and body weight. *** *p* < 0.001.

**Table 6:** Variations in intestinal protein zonulin levels.

Variable	Treatment	Immediate (Week 0)	Long-Term (Week 4)
Zonulin (ng/mL)	Placebo	4.46 ± 0.85	4.42 ± 0.82
	APS	4.38 ± 0.82	3.81 ± 0.67

Data are presented as means ± SE and comparisons for zonulin.

## Data Availability

The original contributions presented in this study are included in the article. Further inquiries can be directed to the corresponding author.

## Supplementary Materials

The following supporting information can be downloaded at: https://www.mdpi.com/article/10.3390/nu18030504/s1; Table S1: Two-Way Repeated-Measures ANOVA for changes in muscle function during the 4-week intervention; Table S2: Two-way repeated-measures ANOVA for changes in intestinal protein zonulin levels; Table S3: Safety evaluation in APS and Placebo groups across the intervention.

## Abbreviations

The following abbreviations are used in this manuscript:

APS	*Astragalus membranaceus* and *Panax notoginseng saponins*
EAAs	Essential amino acids
AUC	Area under the curve
CAT	Cationic amino acid
BUN	Blood urea nitrogen
AST	Aspartate aminotransferase
ALT	Alanine aminotransferase
MPS	Muscle protein synthesis
BCAAs	Branched-chain amino acids
BMI	Body mass index
mTOR	Mammalian target of the rapamycin
