# Supplementary material for: Effects of Astragalus membranaceus and Panax notoginseng Saponins Extract on the Pharmacokinetics of Whey Protein Absorption, Intestinal Permeability, and Muscle Function: A Pilot Study
Source: Nutrients. 2026 Feb 2;18(3):504. doi: 10.3390/nu18030504 (PMC12899613; doi:10.3390/nu18030504)
Supplement: Supplementary file 1 [file nutrients-18-00504-s001.zip › nutrients-4129015-supplementary.pdf]

Table S1. Two-Way Repeated-Measures ANOVA for changes in muscle function during the 4-week intervention.

Variable	Source of variation	F (1, 29)	P value
Grip Strength (kg)	Treatment	3.559	0.069
	Time	11.648	0.002**
	Treatment × Time	2.072	0.161
Muscle Mass (kg)	Treatment	2.278	0.142
	Time	0.031	0.863
	Treatment × Time	1.297	0.264
Body Weight (kg)	Treatment	0.011	0.917
	Time	1.471	0.235
	Treatment × Time	0.805	0.377

**$p < 0.01$.

Table S2. Two-way repeated-measures ANOVA for changes in intestinal protein zonulin levels.

Variable	Source of variation	F (1, 29)	P value
Zonulin (ng/mL)	Treatment	1.962	0.174
	Time	0.472	0.498
	Treatment × Time	0.364	0.552

Table S3. Safety evaluation in APS and Placebo groups across the intervention.

Variable	Treatment	Immediate (week 0)	Long-term (week 4)	p-value
BUN (mg/dL)	Placebo	11.90±0.79	15.37±0.84	<0.01
	APS	12.87±0.63	15.90±0.92	<0.01
Creatinine (mg/dL)	Placebo	0.75±0.03	0.73±0.03	0.182
	APS	0.73±0.03	0.72±0.03	0.448
AST (U/L)	Placebo	20.63±1.24	19.47±1.48	0.177
	APS	19.13±1.69	19.33±2.13	0.933
ALT (U/L)	Placebo	18.43±1.88	19.87±2.46	0.396
	APS	18.17±2.03	20.83±4.67	0.532

Data are presented as means ± SE and post-hoc comparisons for Bun, Creatinine, AST, and ALT.
